# Prognostic Value of Lactate Dehydrogenase in Patients with Hepatocellular Carcinoma: A Meta-Analysis

**DOI:** 10.1155/2018/1723184

**Published:** 2018-12-27

**Authors:** Weihao Kong, Xiaomin Zuo, Hao Liang, Jingxiong Hu, Huabing Zhang, Xingyu Wang, Wei Chen

**Affiliations:** ^1^Department of Emergency Surgery, Department of Emergency Medicine, The First Affiliated Hospital of Anhui Medical University, Heifei, China; ^2^Department of General Surgery, The First Affiliated Hospital of Anhui Medical University, Hefei, China; ^3^Department of Hepatobiliary Surgery, The Third affiliated hospital of Sun Yat-sen University, Guangzhou, China; ^4^Department of Biochemistry & Molecular Biology, School of Basic Medicine, Anhui Medical University, Heifei, China

## Abstract

**Background:**

Previous studies have shown the prognostic value of lactate dehydrogenase (LDH) in hepatocellular carcinoma (HCC), but the results are not persuasive. Therefore, the purpose of our study was to quantitatively explore the prognostic value of LDH in hepatocellular carcinoma.

**Methods:**

We searched the Web of Science, Embase, PubMed, and the Cochrane Library for literature published before October 2018 on the prognostic value of LDH in patients with hepatocellular carcinoma. The combined hazard ratios (HRs) and 95% confidence intervals (CIs) were utilized to assess the prognostic value of LDH in overall survival (OS), recurrence-free survival (RFS), and progression-free survival (PFS) of HCC. Subgroup analysis, sensitivity analysis, and metaregression were used to explore the source of heterogeneity. Funnel plots with Begg's test and Egger's test were used to detect potential publication biases. Furthermore, combined odds ratios (ORs) were utilized to assess the correlation between LDH and clinicopathological features.

**Results:**

A total of 10 nonrandomized controlled studies were included in this meta-analysis. The combined effects of LDH on HCC patients' OS, RFS/DFS, and PFS were HR = 2.07, 95% CI: 1.63-2.62, P < 0.001; HR = 1.62, 95% CI: 1.37-1.90, P < 0.001; and HR = 1.96, 95% CI: 1.14-3.36, P = 0.014, respectively. Subgroup analysis and sensitivity analysis showed that the outcome was stable, and the results of the metaregression also identified statistical models as an important source of heterogeneity. Potential publication bias was detected in the OS studies, so the trim-and-fill method was used to explore publication bias, and the results showed stability. Furthermore, the combined OR suggests that LDH was significantly correlated with gender, Child-Pugh grade, alpha-fetoprotein, vascular invasion, and tumor size.

**Conclusions:**

Preoperative LDH elevation is significantly associated with poor prognosis in patients with HCC, which may be a promising factor in assessing the prognosis of patients with HCC.

## 1. Introduction

Hepatocellular carcinoma (HCC) is one of the most common malignancies in the world, and the number of patients increases by more than 1 million per year [1]. Radical resection is currently the best treatment for HCC, but tumor recurrence and distant metastasis after surgery are the biggest problems affecting patient prognosis [2]. At present, the traditional tumor biomarker alpha-fetoprotein (AFP) is used to predict the prognosis of patients with hepatocellular carcinoma, but its sensitivity and specificity are not satisfactory [3, 4]. Therefore, it is crucial to find a valuable novel biomarker for predicting the prognosis of hepatocellular carcinoma.

In our bodies, normal cells obtain energy through aerobic oxidation, but tumor cells obtain energy through glycolysis. Therefore, abnormal activation of the glycolytic pathway is one of the major metabolic transitions during the malignant transformation of tumor cells. Lactate dehydrogenase, an important coenzyme in the glycolytic pathway, can catalyze the conversion of pyruvate to lactic acid and plays an important role in anaerobic glycolysis [5]. LDH has been identified as a valuable tumor marker for poor prognosis in a variety of tumors, including hepatocellular carcinoma [6], gastric cancer [7], breast cancer [8, 9], lung cancer [10], melanoma [11], colorectal cancer [12], thymic cancer [13], gallbladder cancer [14], neuroblastoma [15], and other solid tumors. However, most clinical studies have small sample sizes, and their statistical power is not sufficient to draw convincing conclusions about the prognostic role of elevated LDH levels in HCC patients. Meta-analyses are an extremely useful statistical tool that produce the best estimate of effect size and can address the limitations of sample size differences among multiple studies [16]. Therefore, in this study, we quantitatively evaluated the prognostic role of LDH in patients with HCC by performing a meta-analysis.

## 2. Materials and Methods

### 2.1. Literature Search Strategy

A search was conducted in the electronic databases of the Web of Science, Embase, PubMed, and the Cochrane Library to identify relevant literature (published through October 2018) investigating the association between LDH and HCC. The following terms were the search keywords: “lactate dehydrogenase” OR “LDH” AND “liver cancer” OR “hepatocellular carcinoma” OR “HCC” OR “hepatoma” AND “prognostic” OR “prognosis” OR “outcome” OR “survival”. Only publications in English were included. Two of the authors (Kong WH and Zuo XM) also conducted a manual search to identify potentially eligible studies from references cited in the original studies.

### 2.2. Inclusion and Exclusion Criteria

The inclusion criteria of the selected studies were as follows. (1) The studies were published in English. (2) The studies investigated the association between LDH level and prognosis index including OS, DFS, RFS, and PFS. (3) Patients were divided into two comparable cohorts according to LDH level. (4) The data of HRs or ORs with 95% confidence intervals (CIs) could be calculated. The studies were excluded when they met the following criteria: (1) absence of a cutoff value of LDH in the studies; (2) descriptive text only, without statistical outcomes of interest, including reviews, comments, or case reports; (3) no available data for estimating HRs with 95% CIs or ORs with 95% CIs; and (4) studies unrelated to the topic of interest were removed.

### 2.3. Data Extraction and Quality Assessment

A predefined data extraction form was made by reviewing all candidate publications, which was carried out by two independent investigators (Kong WH and Zuo XM). A third investigator joined to reconcile disagreements when the results were inconsistent. Extracted information included the following: the first author's name, year of publication, region, median age of patients, study design, time of recruitment, follow-up, number of patients, stage range of HCC, cutoff value of LDH level, treatment of HCC, HRs with 95% CI of prognosis, and clinicopathological data. The quality of each study was assessed by two independent investigators, based on the Newcastle-Ottawa Quality Assessment Scale (NOS) and the Risk Of Bias In Nonrandomized Studies of Exposures (ROBINS-E) tool [17]. A study with a NOS score of 6 or more was defined as a high-quality study [18]. The ROBINS-E tool evaluated the bias of the included studies. In addition, we registered the meta-analysis on PROSPERO website (https://www.crd.york.ac.uk/PROSPERO, registration number: CRD42018114269).

### 2.4. Statistical Analysis

The meta-analysis was performed using Stata SE12.0 (StataCorp, College Station, TX). HRs and their 95% CIs were aggregated to assess the effect of elevated LDH levels on prognosis. When HRs and 95% CIs were not directly reported in some studies but the Kaplan-Meier curves were provided for OS, DFS, RFS or PFS, the Engauge Digitizer version 4.0 (http://digitizer.sourceforge.net/) software was applied to extract the survival data. When the prognostic analysis data were provided with both univariate and multivariate analyses, only the latter was extracted. To evaluate the association between LDH levels and clinicopathological characteristics, ORs and their 95% CIs were calculated. HR > 1 implied that patients with elevated LDH levels had a worse prognosis, and OR > 1 indicated that patients with elevated LDH levels had unfavorable clinicopathological characteristics. The results were considered statistically significant when the P value was less than 0.05. Chi-squared tests and inconsistency index (I^2^) statistics were applied to assess the heterogeneity of the studies. I^2^ >50% or P<0.05 indicated statistical heterogeneity. When statistical heterogeneity did not exist, we used a fixed effects model to assess the pooled HRs. Otherwise, random effects models were employed. Sensitivity analysis was conducted by removing each study individually to evaluate the stability of results in this meta-analysis. Funnel plots with Begg's test and Egger's test were used to detect the potential publication bias. An asymmetry of the funnel plot with a P value of < 0.05 was regarded as a significant publication bias.

## 3. Results

### 3.1. Literature Search

**Figure 1:**
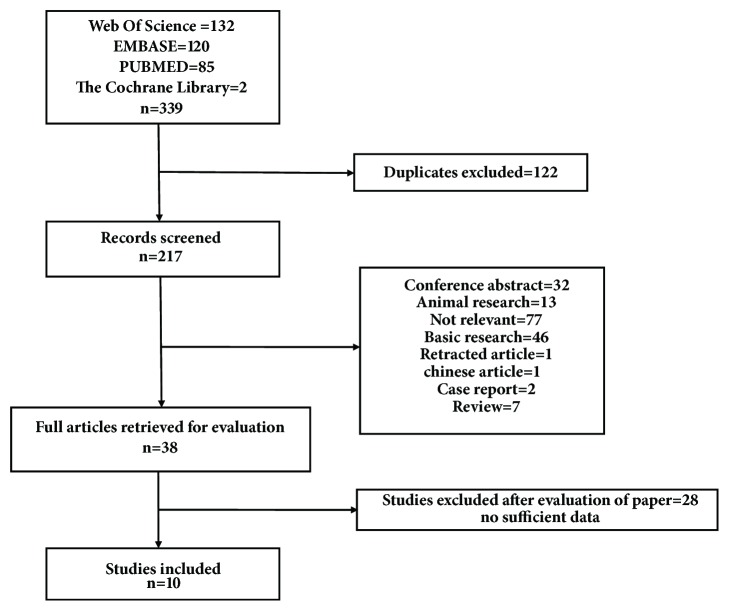
Flow chart of the study selection.

After a systematic search of the electronic databases, 339 articles were identified (the Web of Science=132, Embase=120, Pubmed=85, and the Cochrane Library=2). The detailed search strategy is shown in supplementary file 1. A total of 122 duplicates were removed, leaving 217 articles for further selection. After removing the irrelevant articles, conference abstracts, animal research, basic research, retracted articles, case reports, and reviews, the remaining 38 articles were evaluated by full text reading. Ultimately, 10 eligible studies were included in the present meta-analysis. Details of the study screening process are presented in Figure 1.

### 3.2. Study Characteristics

The basic characteristics of the 10 selected studies from 2010 to 2016 are summarized in Table 1. All included studies were retrospective studies that were published in English. A total of 2576 patients were included in the meta-analysis. The sample size of the selected studies ranged from 37 to 743. The recruitment of patients was carried out from 2000 to 2014. The stage of HCC was evaluated by two methods in the included studies, including BCLC and TNM stage. All of the included studies reported their cutoff of LDH expression, which was diverse across studies. The therapies included curative resection, transcatheter arterial chemoembolization (TACE), chemotherapy, radiofrequency ablation (RFA), percutaneous ethanol injection therapy (PEIT), hepatic artery infusion chemotherapy (HAIC), radiation therapy (RT), best supportive care (BSC), and transcatheter arterial infusion chemotherapy (TAI). Among the ten studies, the NOS score of five studies exceeded 6, and the detailed contents of the ROBINS-E table are shown in supplementary file 2. Nine studies reported OS, while three studies reported PFS. However, because only two studies reported RFS and one study reported DFS, we combined the RFS and DFS to calculate HR with 95% CI.

### 3.3. Correlation of LDH Level with OS in HCC

**Figure 2:**
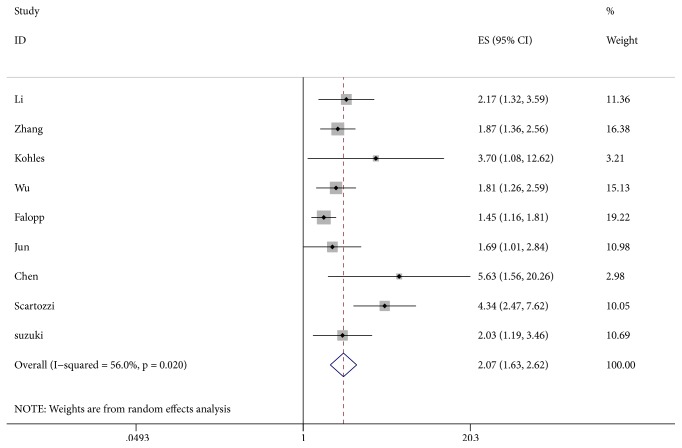
Results of the combined hazard ratio for OS in HCC patients with elevated LDH levels. Notes: OS: overall survival; LDH: lactate dehydrogenase.

The LDH levels and OS were reported in 9 studies [6, 19–26]. A random effect model was utilized to calculate pooled HR due to severe heterogeneity (I^2^ = 56.0%, P = 0.020). The relationship between elevated serum LDH levels and OS is shown in Figure 2. Our results demonstrated that elevated serum LDH levels were significantly correlated with worse OS (HR = 2.07, 95% CI: 1.63-2.62, P < 0.001).

The heterogeneity across the studies was further investigated by subgroup analyses. Subgroup analyses were conducted based on sample size, region, cutoff values, tumor stage classification, and therapy method. The results are presented in Table 2. Additionally, we found that elevated serum LDH was correlated with poor OS in the subgroup of sample size (<100 or ≥100) (HR = 2.11, 95% CI: 1.29-3.46, P = 0.003 or HR = 2.13, 95% CI: 1.61-2.81, P < 0.001). The same results were found in the subgroup of region (Asian or Caucasian) (HR = 1.93 or 2.67, 95% CI: 1.60-2.32 or 1.11-6.44, P < 0.001 or P = 0.029), tumor stage classification (BCLC, TNM or others) (HR = 2.11, 1.82 or 2.73, 95% CI: 1.40-3.17, 1.39-2.38 or 1.53-4.89, P < 0.001, P < 0.001 or P = 0.001), and therapy method (resection, TACE or others) (HR = 1.95, 4.22 or 1.61, 95% CI: 1.44-2.06, 2.53-7.04 or 1.35-1.93, P < 0.001, P < 0.001 or P < 0.001). These results were in accordance with the outcome of pooled OS. However, in the subgroup of cutoff values, no statistical significance was detected when the cutoff value was ≤200 (HR = 2.70, 95% CI: 0.97-7.50, P =0.057), but cutoff values of 200-400 (HR = 2.00, 95% CI: 1.56-2.57, P < 0.001) and ≥400 (HR = 2.12, 95% CI: 1.14-3.94, P = 0.018) were significant. Furthermore, we performed a meta-regression and found that the statistical model was correlated with the heterogeneity between the studies, which can explain 85.88% of the heterogeneity (supplementary file 3).

### 3.4. Correlation of LDH Level with RFS/ DFS in HCC

**Figure 3:**
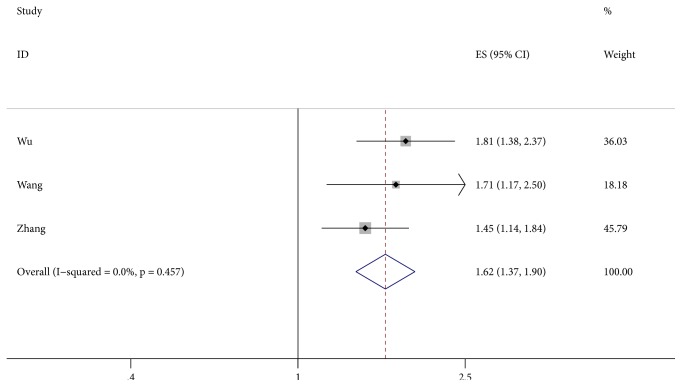
Results of the combined hazard ratio for RFS/DFS in HCC patients with elevated LDH levels. Notes: RFS: recurrence-free survival; DFS: disease free survival; LDH: lactate dehydrogenase.

Three studies [24, 26, 27] provided the data reporting the association between LDH level and RFS/DFS. The pooled HR and 95% CI were calculated with a fixed-effect model because no significant heterogeneity was detected (I^2^ = 0.0%, P = 0.457). We found that elevated LDH levels were significantly correlated with worse RFS/DFS (HR = 1.62, 95% CI: 1.37-1.90, P < 0.001) (Figure 3).

### 3.5. Correlation of LDH Level with PFS in HCC

**Figure 4:**
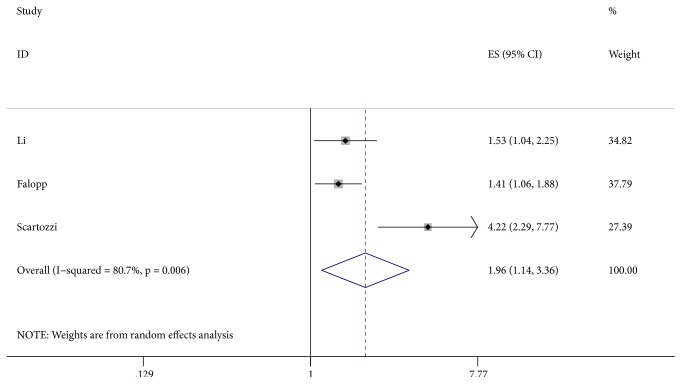
Results of the combined hazard ratio for PFS in HCC patients with elevated LDH levels. Notes: PFS: progression-free survival; LDH: lactate dehydrogenase.

Three studies [6, 21, 25] described the relationship between LDH level and PFS. Our meta-analysis with a random effect model (I^2^ = 80.7%, P = 0.006) showed that elevated LDH levels were correlated with poor PFS (HR = 1.96, 95% CI: 1.14-3.36, P = 0.014) (Figure 4).

### 3.6. Publication Bias and Sensitivity Analysis

**Figure 5:**
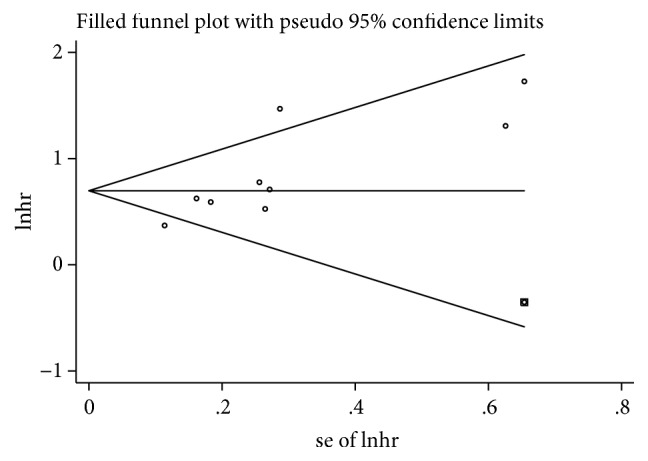
Funnel plot to evaluate the publication bias for OS. Notes: OS: overall survival.

**Figure 6:**
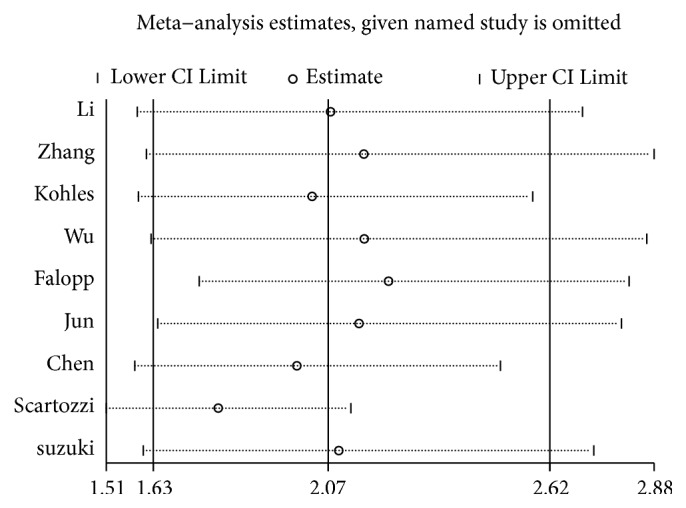
Sensitivity analysis of combined HR for OS. Notes: HR: hazard ratio; OS: overall survival.

The potential publication bias of the studies included in the OS analysis was evaluated by funnel plot and Egger's test. The shape of the funnel plots with Begg's test and Egger's test indicated existent asymmetry for OS (Begg's test: P = 0.029, Egger's test: P = 0.010). Then, the trim-and-fill analysis was adopted by adding one missing study (Figure 5), and the results showed that the outcome before and after the trim-and-fill method was stable. Sensitivity analyses were conducted to estimate the stability of each study on the pooled results of the OS. From the results of the sensitivity analyses, no significant influence was detected after removing any single study, which indicated that our conclusions were reliable (Figure 6). Considering the small amount of literature, we did not carry out the publication bias or sensitivity analysis of RFS/DFS and PFS.

### 3.7. Correlation of LDH Level with Clinicopathological Factors in HCC

The results of the association between elevated LDH levels and clinicopathological parameters are described in Table 3. The results of the pooled analysis revealed that elevated LDH levels were correlated with gender (male versus female) (OR = 0.68, 95% CI: 0.49-0.95, P = 0.025), worse Child-Pugh grade (B versus A) (OR = 1.90, 95% CI: 1.47-2.45, P < 0.001), higher AFP levels (high versus low) (OR = 1.52, 95% CI: 1.26-1.82, P < 0.001), vascular invasion (yes versus no) (OR = 1.59, 95% CI: 1.36-1.86, P < 0.001), advanced TNM stage (III+IV versus I+II) (OR = 1.62, 95% CI: 1.28-2.05, P < 0.001), and larger tumor diameter (>5 cm versus <5 cm) (OR = 2.03, 95% CI: 1.23-3.35, P = 0.006). However, no statistical significance was observed for age (old versus young) (OR = 1.19, 95% CI: 0.99-1.45, P = 0.070), ECOG score (1+2 versus 0) (OR = 1.22, 95% CI: 0.88-1.69, P = 0.232) or tumor number (multiple versus single) (OR = 1.25, 95% CI: 0.99-1.58, P = 0.063).

## 4. Discussion

Currently, many studies have focused on finding tumor markers to predict cancer prognosis. For HCC, researchers have found that elevated levels of many tumor markers are associated with poor prognosis in patients with HCC, such as alpha-fetoprotein, neutrophil to lymphocyte ratio, and platelet to lymphocyte ratio [28, 29]. Although researchers have long studied the relationship between LDH and the prognosis of patients with HCC, the prognostic role of LDH in HCC is still not conclusive. Therefore, we conducted a meta-analysis of published literature to combine the statistical effects of LDH and the prognosis of patients with hepatocellular carcinoma.

In this meta-analysis, a total of 10 studies explored the prognostic role of LDH in patients with hepatocellular carcinoma. The statistics of OS, PFS, and RFS/DFS related to LDH levels in the studies were subsequently combined. Our meta-analysis confirmed that elevated LDH is a risk factor for OS, PFS, and RFS/DFS in HCC patients. In addition, due to the limited number of studies, we only performed a subgroup analysis, a sensitivity analysis, and publication bias detection on the OS. The results of the subgroup analysis showed that elevated levels of LDH were significantly associated with poor prognosis in patients with HCC in the sample size, ethnic origin, and tumor staging classification subgroup. However, when analyzing the cutoff subgroup of OS, LDH levels below 200 were not significantly associated with the prognosis of patients with hepatocellular carcinoma, which can be explained by the fact that when the value of LDH falls within the normal range, there is no significant difference from healthy people. The potential publication bias of the OS study was detected by Begg's test and Egger's test, so a study was added by the trim-and-fill method, and the results showed that the combined HR was stable. Moreover, the results of the sensitivity analysis also showed that the elimination of any study had no significant effect on the overall outcome, so the results of our meta-analysis were reliable and convincing. At the same time, the results of the metaregression indicate that the statistical model is an important source of heterogeneity. Finally, the results of the combined OR showed that LDH was significantly correlated with gender, Child-Pugh grade, AFP, vascular invasion, and tumor size. AFP, vascular invasion, and tumor size have been confirmed to be significantly correlated with the prognosis of patients with HCC, thus explaining to some extent why the prognosis of HCC patients with elevated LDH levels is worse.

Several potential mechanisms have been explored that may explain the link between elevated LDH levels and hepatocellular carcinoma progression. First, tumor cells regulate the important coenzymes in the glycolytic pathway, such as LDH, through the AMPK signaling pathway to provide energy for tumor cell proliferation and progression [30]. Second, Zhai et al. found that oxalic acid induced on inhibitory effect on LDH by downregulating the cyclin-dependent kinase 1/cyclin B1 pathway, which led to G2/M cell cycle arrest and promoted tumor cell apoptosis by enhancing the effects of mitochondrial reactive oxygen species (ROS) [31]. Third, an increase in LDH activity promotes the accumulation of lactic acid, which in turn causes a decrease in the pH of the matrix surrounding the tumor cells and leads to increased macrophage-mediated angiogenesis [32, 33]. Moreover, mitochondria can also be protected from oxidative stress in an acidic environment with low pH, thereby enhancing the resistance of tumor cells to hypoxia-induced apoptosis [34]. Finally, LDH5, an important isozyme of LDH, plays a crucial role in the invasive phenotype of tumor cells through the expression of hypoxia-inducible factors (HIF) and vascular endothelial growth factor (VEGF) [35].

Therefore, LDH can be used as an indicator of prognosis in hepatocellular carcinoma. Compared with the traditional tumor biomarker of hepatocellular carcinoma, AFP, LDH has the advantages of low cost, simple operation, and rapid detection. When we reviewed some published literature concerning multivariate analyses and differential weight for these two factors (LDH and AFP), we found that the weight of LDH is larger than that of AFP in most of the literature. In a study of overall survival conducted by Li, Zhang, Kohles, and Wu [24–26], the weight of LDH was greater than that of AFP, while, in the study of recurrence-free survival conducted by Wu and Wang [26, 27], the weight of AFP was greater than that of LDH. This may indicate that the value of LDH is superior to that of AFP in predicting overall survival in patients with hepatocellular carcinoma, whereas AFP is superior to LDH in predicting recurrence-free survival in patients with hepatocellular carcinoma.

At the same time, our meta-analysis has some limitations that need attention. First, although we used a random effects model to combine the effects, there was still considerable heterogeneity. Nevertheless, the results of the subgroup analysis and sensitivity analysis are in agreement with the overall effect, so our results are stable and credible. Second, there is a publication bias in our research. This bias most likely occurred because we only included studies published in English, and unpublished and non-English literature was ignored. In addition, people are more likely to publish positive results than negative results, which can cause a certain bias. Therefore, we used the trim-and-fill method to analyze the included studies, and the conclusions did not change significantly. The results of the sensitivity analysis also showed that the results were stable, so our conclusions are credible. Third, it is difficult to define the optimal cutoff value for LDH. On the one hand, the sample sizes were different in the included studies; on the other hand, the exposure factors for each study were different. Similarly, race, region, and detection method also affect the cutoff value for LDH. Therefore, determining an optimal cutoff value is very difficult, so we need more large-scale multicenter studies to determine the optimal cutoff value for LDH. Fourthly, since our protocol is retrospectively registered, it may have an impact on the repeatability and credibility of the study. Finally, the HR of some studies was extracted from the survival curve, which may deviate from the actual results.

## 5. Conclusion

Despite the limitations of the research, we can still draw some valuable conclusions. Our meta-analysis demonstrates that elevated levels of LDH are significantly associated with poor prognosis in patients with HCC. LDH is also significantly associated with gender, Child-Pugh grade, AFP, vascular invasion, and tumor size. Hence, LDH can be used as a potential marker to predict the prognosis of patients with HCC, thus providing new information for the prevention and treatment of HCC. However, a large sample of multicenter studies is needed in the future to confirm the conclusion of our meta-analysis.

## Figures and Tables

**Table 1:** Main characteristics of the included studies.

First author	Year	Region	Age, median (range)	Cancer type	Study design	Time of Recruitment	Follow-up (months)	Number of patients	Stage range	Cutoff value	Therapy	Outcome	statistical mode	NOS score
Li	2016	China	54(19-79)	HCC	Retrospective	2010.1-2014.12	15(3-73)	119	BCLC(BC)	221	TACE/Chemotherapy	OS/PFS	M	7
Zhang	2015	China	NA	HCC	Retrospective	2008.1-2012.6	41(1-86)	683	TNM(I-IV)	188	curative resection	OS/DFS	M	7
Kohles	2012	Germany	66.7(mean)	HCC	Retrospective	2006-2008	Max:24	38	NA	201	TACE	OS	M	6
Wu	2016	China	48(17-81)	HCC	Retrospective	2007.6-2013.3	42(2-99)	469	BCLC(0ABC)	203.5	curative resection	OS/RFS	M	7
Falopp	2014	Italy	NA	HCC	Retrospective	2008-2012	Max:50	78	BCLC(BC)	407	sorafenib	OS/PFS	M	7
Jun	2013	Korea	59.1(32-87)	HCC	Retrospective	2005.1-2012.12	27.3(0-115)	743	TNM(I-IV)	450	surgical resection/PEIT/RFA/TACE/HAIC/ RT/BSC	OS	M	6
Wang	2015	China	54±12.1	HCC	Retrospective	2007.5-2011.5	39.4(3-97.6)	200	BCLC(0ABC)/TNM(I-IV)/ Edmonson-Steiner grade (I-IV)	206	curative resection	RFS	M	6
Chen	2010	China	53.15±11.63	HCC	Retrospective	2001.1-2007.12	Max:2500 days	37	NA	200	surgery after admission	OS	M	6
Scartozzi	2012	Italy	NA	HCC	Retrospective	2002-2010	Max:30	114	BCLC(ABC)	450	TACE	OS/PFS	U	7
suzuki	2015	Japan	68(37-82)	HCC	Retrospective	2000.2-2010.10	Max:80	95	NA	230	TAI	OS	M	6

Notes: NOS: Newcastle-Ottawa Scale; NA: not available; HCC: hepatocellular carcinoma; BCLC: Barcelona Clinic Liver Cancer; TNM: Tumor Node Metastasis; Max: maximum; TACE: transcatheter arterial chemoembolization; RFA: radiofrequency ablation; PEIT: percutaneous ethanol injection therapy; HAIC: hepatic artery infusion chemotherapy; RT: radiation therapy; BSC: best supportive care; TAI: transcatheter arterial infusion chemotherapy; OS: overall survival; RFS: recurrence-free survival; DFS: disease free survival; PFS: progression-free survival; M: multivariate analysis; U: univariate analysis.

**Table 2:** Subgroups analysis of combined HR for OS in HCC patients.

Subgroups	NO. of studies	NO. of patients	Pooled HR	P-value	Heterogeneity		
			(95% CI)		I^2^ (%)	P-value	Model
Sample size							
<100	4	248	2.11 (1.29-3.46)	**0.003**	57.2	0.071	random
≥100	5	2128	2.13 (1.61-2.81)	**0.000**	51.4	0.084	random
Region							
Asian	6	2146	1.93 (1.60-2.32)	**0.000**	0	0.646	random
Caucasian	3	230	2.67 (1.11-6.44)	**0.029**	85.9	0.001	random
Cutoff value							
≤200	2	720	2.70 (0.97-7.50)	0.057	62.8	0.101	random
200-400	4	721	2.00 (1.56-2.57)	**0.000**	0	0.710	random
≥400	3	935	2.12 (1.14-3.94)	**0.018**	84.2	0.002	random
Tumor stage type							
BCLC	4	780	2.11 (1.40-3.17)	**0.000**	77.8	0.004	random
TNM	2	1426	1.82 (1.39-2.38)	**0.000**	0	0.754	random
Others	3	170	2.73 (1.53-4.89)	**0.001**	20.6	0.284	random
Therapy method							
resection	3	1189	1.95 (1.44-2.06)	**0.000**	29.7	0.241	random
TACE	2	152	4.22 (2.53-7.04)	**0.000**	0	0.817	random
others	4	1035	1.61 (1.35-1.93)	**0.000**	0	0.392	random

Notes: HR: hazard ratio; 95% CI: 95% confidence intervals; OS: overall survival; NO: number; BCLC: Barcelona Clinic Liver Cancer; TNM: Tumor Node Metastasis; TACE: transcatheter arterial chemoembolization.

**Table 3:** Meta-analysis of the correlation between LDH and clinicopathological features in HCC patients.

Stratified analysis	No. of studies	No. of patients	Pooled OR	p-value	Heterogeneity		
			(95% CI)		I^2^ (%)	P-value	Model
Age (old vs. young)	3	875	1.19 (0.99-1.45)	0.070	0	0.894	Fixed

Gender	5	1463	0.68 (0.49-0.95)	**0.025**	67.2	0.016	random
(male vs. female)							

ECOG score	3	311	1.22 (0.88-1.69)	0.232	33.8	0.221	fixed
(1+ 2 vs. 0)							

Tumor number	3	1271	1.25 (0.99-1.58)	0.063	51.1	0.129	random
(multiple vs. single)							

Child-Pugh Grade	3	916	1.90 (1.47-2.45)	**0.000**	0	0.951	fixed
(B vs. A)							

AFP (high vs. low)	2	547	1.52 (1.26-1.82)	**0.000**	0	0.384	fixed

Vascular invasion	3	1271	1.59 (1.36-1.86)	**0.000**	12.9	0.317	fixed
(yes vs. no)							

TNM stage	2	802	1.62 (1.28-2.05)	**0.000**	0	0.875	fixed
(III+IV vs. I+II)							

Tumor diameter	2	1152	2.03 (1.23-3.35)	**0.006**	89.9	0.002	random
(> 5vs. <5)							

Notes: LDH: lactate dehydrogenase; NO: number; OR: odds ratio; 95% CI: 95% confidence intervals; ECOG: Eastern Cooperative Oncology Group; AFP: alpha-fetoprotein; TNM: Tumor Node Metastasis.

## Data Availability

The detailed data used to support the findings of this study have been deposited in the Open Science Framework (osf.io) repository (DOI 10.17605/OSF.IO/AX72F), which included selection of included studies, stata commands for statistical analysis, and stata data.

## Supplementary Materials

Supplementary 1 Supplementary  file  1. Search strategy for the meta-analysis.

Supplementary 2 Supplementary  file  2. The Risk Of Bias In Nonrandomized Studies of Exposures (ROBINS-E) tool was utilized to evaluate publication bias in the observational studies.

Supplementary 3 Supplementary  file  3. Metaregression of combined HR for OS. Notes: HR: hazard ratio; OS: overall survival.
