# Supplementary material for: Prognostic Value of Lactate Dehydrogenase in Patients with Hepatocellular Carcinoma: A Meta-Analysis
Source: Biomed Res Int. 2018 Dec 27;2018:1723184. doi: 10.1155/2018/1723184 (PMC6327280; doi:10.1155/2018/1723184)
Supplement: Supplementary 1 — Supplementary file 1. Search strategy for the meta-analysis. [file 1723184.f1.docx]

Pubmed:

(((("prognostic"[Title/Abstract] OR "prognosis"[Title/Abstract] OR "outcome"[Title/Abstract] OR "survival"[Title/Abstract]))) AND (("Lactate Dehydrogenase"[Title/Abstract]) OR "LDH"[Title/Abstract])) AND (((("hepatoma"[Title/Abstract]) OR "HCC"[Title/Abstract]) OR "liver cancer"[Title/Abstract]) OR "hepatocellular carcinoma"[Title/Abstract]) 85

Embase:

No. Query Results Results Date

#3. 'outcome':ti,ab OR 'survival':ti,ab OR 2,727095 28 Oct 2018

'prognosis':ti,ab OR 'prognostic':ti,ab

#2. 'liver cancer':ti,ab OR 'hepatocellular 130806 28 Oct 2018

carcinoma':ti,ab OR 'hcc':ti,ab

#1. 'lactate dehydrogenase':ti,ab OR 'ldh':ti,ab 73,344 28 Oct 2018

#4. #1 AND #2 AND #3 120 28 Oct 2018

Web of science

# 1主题: ("prognostic" OR "prognosis" OR "outcome" OR "survival") 1,485,849

索引=SCI-EXPANDED, CCR-EXPANDED, IC 时间跨度=所有年份

# 2 主题: ("Lactate Dehydrogenase" OR "LDH")

索引=SCI-EXPANDED, CCR-EXPANDED, IC 时间跨度=所有年份 31,652

# 3 主题: ("HCC" OR "liver cancer" OR "hepatoma" OR "hepatocellular carcinoma")

索引=SCI-EXPANDED, CCR-EXPANDED, IC 时间跨度=所有年份 115,094

# 4 #3 AND #2 AND #1 132
