# Supplementary material for: Prognostic Value of Lactate Dehydrogenase in Patients with Hepatocellular Carcinoma: A Meta-Analysis
Source: Biomed Res Int. 2018 Dec 27;2018:1723184. doi: 10.1155/2018/1723184 (PMC6327280; doi:10.1155/2018/1723184)
Supplement: Supplementary 2 — Supplementary file 2. The Risk Of Bias In Nonrandomized Studies of Exposures (ROBINS-E) tool was utilized to evaluate publication bias in the observational studies. [file 1723184.f2.doc]

| study | pre-exposure | | At exposure | Post-exposure | | | | Overall bias |
| --- | --- | --- | --- | --- | --- | --- | --- | --- |
|  | Bias due to confounding | Bias in selection of participants into the study | Bias in classification of exposures | Bias due to departures from intended exposures | Bias due to missing data | Bias in measurement of outcomes | Bias in selection of the reported result |  |
| Li | Low | Moderate | Low | Moderate | Low | Low | Low | Moderate |
| Zhang | Low | Moderate | Low | Low | Low | Low | Low | Moderate |
| Kohles | High | Moderate | Low | Moderate | Low | Low | Low | High |
| Wu | Low | Moderate | Low | Low | Low | Low | Low | Moderate |
| Falopp | Low | Moderate | Low | Low | Low | Low | Low | Moderate |
| Jun | High | Moderate | Low | Moderate | Low | Low | Low | High |
| Wang | High | Moderate | Low | Low | Low | Low | Low | High |
| Chen | Moderate | Moderate | Moderate | Low | Moderate | Low | Low | Moderate |
| Scartozzi | Moderate | Moderate | Low | Low | Low | Low | Low | Moderate |
| suzuki | Moderate | Moderate | High | Low | Low | Low | Low | High |

Supplementary file2. The Risk Of Bias In Non-randomized Studies - of Exposures (ROBINS-E) tool was utilized to evaluate publication bias in the observational studies.
