# Supplementary figures and images for: Prognostic Value of Lactate Dehydrogenase in Patients with Hepatocellular Carcinoma: A Meta-Analysis
Source: Biomed Res Int. 2018 Dec 27;2018:1723184. doi: 10.1155/2018/1723184 (PMC6327280; doi:10.1155/2018/1723184)

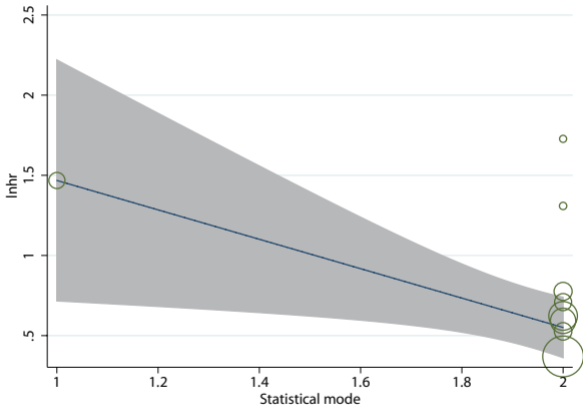

Supplement: Supplementary 3 — Supplementary file 3. Metaregression of combined HR for OS. Notes: HR: hazard ratio; OS: overall survival. [file 1723184.f3.pdf]
